# Spherical V-Fe-MCM-48: The Synthesis, Characterization and Hydrothermal Stability

**DOI:** 10.3390/ma8041752

**Published:** 2015-04-14

**Authors:** Wang Qian, Haiqing Wang, Jin Chen, Yan Kong

**Affiliations:** State Key Laboratory of Materials-Oriented Chemical Engineering, College of Chemistry and Chemical Engineering, Nanjing Tech University, Nanjing 210009, China; E-Mails: wqian@njtech.edu.cn (W.Q.); hiking@njtech.edu.cn (H.W.); okachen30@gmail.com (J.C.)

**Keywords:** mesoporous sieve, MCM-48, co-doping, morphology, hydrothermal stability

## Abstract

Spherical MCM-48 mesoporous sieve co-doped with vanadium and iron was successfully synthesized via one-step hydrothermal method. The material was characterized by X-ray diffraction (XRD), nitrogen adsorption-desorption isotherms, inductively coupled plasma (ICP), scanning electron microscopy (SEM), transmission electron microscopy (TEM), diffuse reflectance UV-vis spectra, and X-ray photoelectron spectra (XPS) techniques. Results indicated that the V-Fe-MCM-48 showed an ordered 3D cubic mesostructure with spherical morphology, narrow pore size distribution and high specific surface area. Most of vanadium and iron atoms existing as tetrahedral V^4+^ and Fe^3+^ species were co-doped into the silicate framework. The particle sizes of V-Fe-MCM-48 were smaller and the specific area was much higher than those of of V-MCM-48. Additionally, the synthesized V-Fe-MCM-48 exhibited improved hydrothermal stability compared with the pure MCM-48.

## 1. Introduction

Due to high surface areas, narrow pore size distribution, and tunable pore diameters, the M41S family [1] has been intensely studied and applied to many areas such as catalysis [2], sorption [3] and so on since the first report in 1992. Owing to its three-dimensional (3D) cubic mesophase with gyroidal structure, MCM-48 favors much more to the mass transfer than MCM-41 [4,5,6,7] as the latter only shows the two-dimensional (2D) hexagonal mesophase [8,9]. Particularly, MCM-48 is more resistant to the pore-blocking [10].

In heterogeneous catalysis, the diffusion of reactants and products highly depend on the pore structure and morphology of catalyst [10]. For example, MCM-41 with various morphologies have been widely reported, e.g., sphere, rod, tube, *etc.* [11,12,13]. By contrast, less attention has been paid to the synthesis of MCM-48 with high quality and special morphology. One of the reasons is that the experimental conditions, including pH, temperature, stirring rate, stirring time, aging time as well as concentration of the template, have to be controlled precisely in order to the obtain materials of desirable mesostructure and defined morphology. In most of cases, the minor changes in the synthesizing conditions may cause disastrous effect to the final product [10,14].

Due to the lack of acidity/basicity and redox properties, pure mesoporous silicate materials show limited applications in the field of catalysis. Therefore, to construct catalytic active sites, considerable effort has been spent in incorporating transition metals like Ti, V, Fe, Cu, Ce and so on [4,14,15,16,17] into the silicate framework by a variety of methods, which include one-step hydrothermal method [4,18], wet impregnation method [19], template ion-exchange method [6] and grafting method [20]. However, many methods are not desirable as they are relatively easier to induce the formation of crystalline metal oxides outside of the framework, which may result in the elution of catalytic active sites in the liquid-phase reactions. By contrast, the one-step hydrothermal method is more advantageous in this aspect because of its facile synthesis procedures and remarkable capability of incorporating heteroatoms into the framework [19,21,22].

Furthermore, the relatively poor hydrothermal stability also limits the application of mesoporous silicate materials in the liquid-phase reactions [23]. Some approaches to improve the hydrothermal stability have been reported, such as incorporating heteroatoms [24,25,26], pH adjusting [27], addition of crystal seed precursors, inorganic salts, or PVA powder [28,29,30,31].

In the cases of the incorporations of metals into the materials, the bimetallic catalyst shows more potential than monometallic catalyst due to the synergistic effect of bimetal, which may improve the final structure as well as the stability of materials to the great extent. Till now, concerning the synthesis using bimetallic dopants, there have been numerous reports on MCM-41 mesoporous sieves but only sparse reports on the MCM-48. Furthermore, when co-doped with bimetals, it remains even more difficult to obtain materials of special morphology. The precise experimental conditions are therefore demanding for the synthesis of bimetallic MCM-48 with regular morphology. In our previous studies, mesoporous sieves doped with vanadium exhibited high catalytic activity in the oxidation of aromatics, including benzene, styrene and phenol [4,32,33,34,35,36,37]. It was also reported that the incorporation of Fe(III) can enhance the hydrothermal stability of mesoporous sieves [38].

In this study, spherical MCM-48 co-doped with V and Fe was successfully synthesized based upon our work in which spherical V-MCM-48 with the particle size of *ca.* 500 nm was synthesized [4]. The obtained material was further characterized by XRD, N_2_ physisorption, SEM, TEM, ICP, UV-vis, and XPS techniques. The hydrothermal stability of produced V-Fe-MCM-48 was also evaluated.

## 2. Results and Discussion

### 2.1. Mesoporous Structure of V-Fe-MCM-48 Sample

#### 2.1.1. XRD

**Figure 1:**
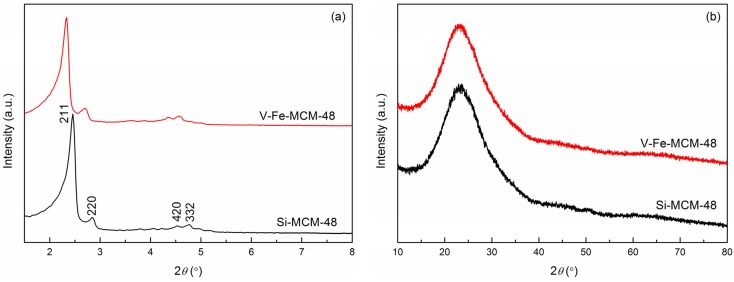
(**a**) Small-angle X-ray diffraction (XRD) patterns and (**b**) wide-angle XRD patterns of the calcined Si- and V-Fe-MCM-48 samples.

The small-angle powder XRD patterns of Si- and V-Fe-MCM-48 samples are shown in Figure 1a. Similar to that of Si-MCM-48, the pattern of V-Fe-MCM-48 exhibits an intense peak of (221) reflection and weak peaks of (220), (332) and (420) reflections, which represent the characteristics of the three-dimensional (3D) cubic mesophase. This result indicates that highly ordered mesostructure of MCM-48 is well retained which is not significantly affected by the presence of dual dopants of V and Fe in the silicate framework. Compared with Si-MCM-48, all of the peaks corresponding to V-Fe-MCM-48 shift evidently to the lower angle region suggesting an increase in unit cell parameter (a_0_) which may be attributed to the replacement of Si^4+^ (Pauling radius = 42 pm) by V^4+^ (Pauling radius = 58 pm) and Fe^3+^ (Pauling radius = 64 pm) [35,39].

The wide-angle XRD pattern of the V-Fe-MCM-48 sample as showed in Figure 1b exhibits a broad peak at 2θ ≈ 23.0°, which is normally ascribed to the characteristic peak of amorphous silica. Beyond that, no characteristic peaks of vanadium oxide and iron oxide are observed suggesting that the sample co-doped with V and Fe atoms still exists as amorphous state without pronounced aggregation and formation of bulk crystals of metal species. We reason that both the V and Fe atoms may be incorporated into the silicate framework or highly dispersed on the surface of mesoporous channels.

#### 2.1.2. Nitrogen Adsorption-Desorption

**Figure 2:**
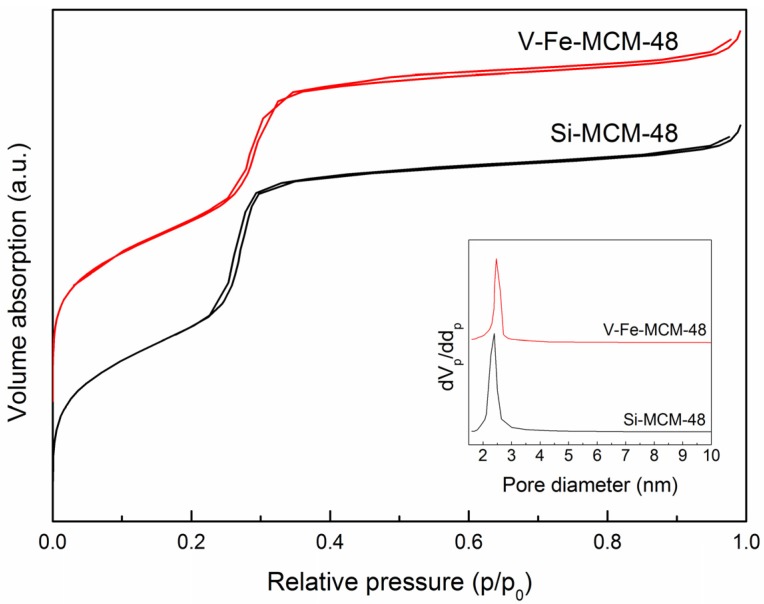
Nitrogen adsorption-desorption isotherms of the calcined Si- and V-Fe-MCM-48 samples. The inset shows the pore size distribution (PSD).

The nitrogen adsorption-desorption isotherms and pore size distribution (PSD) of the calcined Si- and V-Fe-MCM-48 are displayed in Figure 2. It is observed that the V-Fe-MCM-48 sample exhibits a similar type as the Si-MCM-48, which can be classified to the type IV isotherm according to the IUPAC classification [40]. A sharp step at the relative pressure of p/p_0_ in the range of 0.20–0.35 corresponding to the capillary condensation in channel-type mesopores suggests a high quality mesostructure and a narrow pore size distribution (2–3 nm) of obtained sample. However, the step of V-Fe-MCM-48 is less steep than that of Si-MCM-48 and the inflection point of step shifts to a higher relative pressure. This observation indicates that the presence of co-dopants of V and Fe decreases the order of final mesostructure and increases the pore size of materials, which is well consistent to the small-angle XRD results.

Table 1 shows the textural and structural parameters of the calcined Si- and V-Fe-MCM-48 samples. Compared with that of Si-MCM-48, the specific surface area (a_s,BET_) of V-Fe-MCM-48 decreases, which is due to a partial disruption of framework caused by the co-dopants of V and Fe. Nevertheless, it still shows high specific surface area over 1000 m^2^/g. The unit cell parameter (a_0_) increases from 8.80 to 9.33 nm indicating the incorporation of V and Fe in the silicate framework, which is in good agreement with the small-angle XRD results. Notably, the wall thickness (t_w_) exhibits a remarkable increase from 1.65 nm to 1.79 nm. This finding demonstrates that some metal species may be highly dispersed on the surface of mesoporous channels although they escape from the wide-angle XRD detection.

**Table 1:** Textural and structural parameters of the calcined Si- and V-Fe-MCM-48 samples.

Sample	V/Si ^a^	V/Si ^b^	Fe/Si ^a^	Fe/Si ^b^	d_211_	a_0_^c^	d_p_^d^	a_s, BET_	t_w_^e^	V_p_
	(Molar Ratio)	(Molar Ratio)	(Molar Ratio)	(Molar Ratio)	(nm)	(nm)	(nm)	(m^2^/g)	(nm)	(cm^3^/g)
Si-MCM-48	—	—	—	—	3.59	8.80	2.39	1369.4	1.65	0.832
V-Fe-MCM-48	0.0296	0.0066	0.0062	0.0021	3.81	9.33	2.46	1213.2	1.79	0.777

Notes: ^a^ ICP results; ^b^ XPS results; ^c^ Unit cell parameter: a_0_ = 6^1/2^d_211_; ^d^ Pore diameter determined by BJH method using the desorption branch; ^e^ Wall thickness: t_w_ = a_0_/3.0919 − d_p_/2.

#### 2.1.3. SEM and TEM

**Figure 3:**
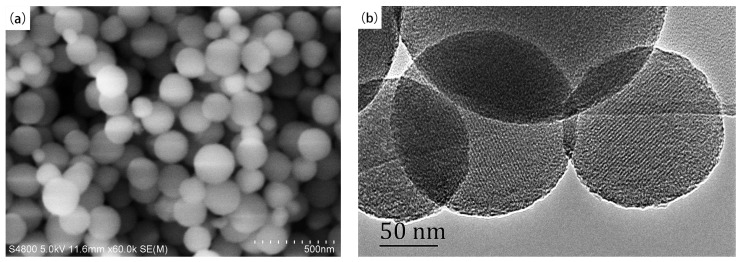
(**a**) Scanning electron microscopy (SEM) image and (**b**) transmission electron microscopy (TEM) image of the calcined V-Fe-MCM-48 sample.

The SEM and TEM images of the calcined V-Fe-MCM-48 sample are displayed in Figure 3. From the SEM image, it is clearly observed that the particles of V-Fe-MCM-48 synthesized in this study show a spherical morphology with most of particles size distribution in the range of 150–250 nm, which is confirmed from the TEM image. It is notably that the introduction of iron species reduced the particle sizes. Meanwhile, the specific area increases significantly from 788 of V-MCM-48 [4] to 1213 m^2^/g of V-Fe-MCM-48 with almost the same amount of vanadium species. Nevertheless, catalyst with higher surface area can be expected more superior activity.

It has been reported that vanadium species mainly exist as HVO_4_^2−^ and V_2_O_7_^4−^ when the pH value is in the range of 10–13 [41]. In our previous report [4], we have ascribed the formation of spherical V-MCM-48 particles to low concentration of V_2_O_7_^4−^ which minimizes the surface free energy, and the particles favors to become irregular in the presence of high concentration of V_2_O_7_^4−^. In the synthesis condition of present work, most of the vanadium species exist as HVO_4_^2−^, which shows lower ionic diameter than that of V_2_O_7_^4−^. Meanwhile, the co-dopant of Fe accelerates the generation of more nuclei, which results in more small crystal growth [42,43,44,45]. In this way, smaller spherical V-Fe-MCM-48 particles are generated.

### 2.2. Statues of Heteroatoms

#### 2.2.1. UV-Vis

**Figure 4:**
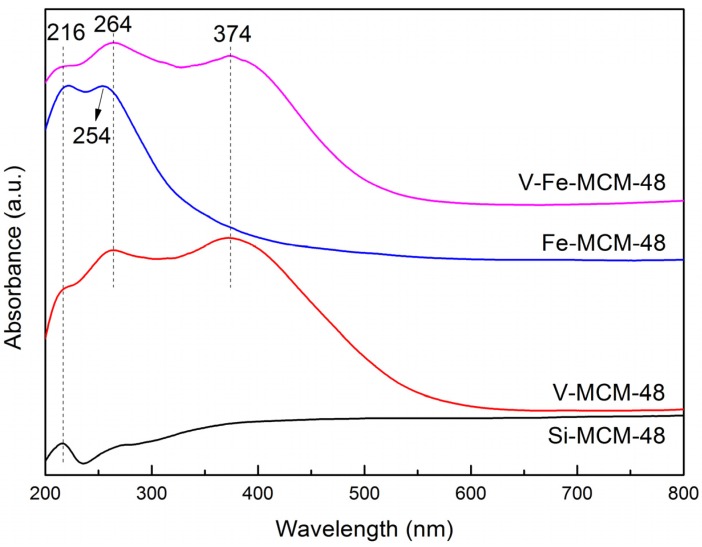
UV-vis spectra of calcined samples.

UV-vis spectra are widely used to investigate the coordination circumstance and oxidation states of metal ions. As shown in Figure 4, all the samples show an absorption band centered at 216 nm, which is attributed to the characteristic absorption of the silica materials [46]. For the calcined V-MCM-48 sample, another two absorption bands are additionally observed in the wavelength range of 220–320 nm and 350–400 nm centered at 264 nm and 374 nm, respectively. The absorption at 264 nm represents the charge-transfer transitions between oxygen ligands and V^4+^ in isolated tetrahedral coordination VO_4_ species, which supports that V atoms have been successfully incorporated into the silicate framework. The band centered at 374 nm is assigned to extra-framework V^5+^ species in distorted octahedral coordination [47]. This observation is possibly due to the interaction between V and water molecules in the atmosphere forming polymeric V-O-V bonds. The band of bulk V_2_O_5_ crystallites (*ca.* 450–480 nm) is absent agreeing well with the wide-angle XRD results. Unlike the calcined V-MCM-48 sample, the calcined 1Fe-MCM-48 sample shows only one absorption peak at 254 nm, which is attributed to the charge-transfer transitions between oxygen ligands and Fe^3+^ in isolated tetrahedral coordination FeO_4_ species [48]. In addition, there is no obvious absorption band at 500–600 nm, which represents octahedral coordination Fe^3+^ species in extra-framework [49]. We conclude that most of Fe^3+^ species are incorporated into the silicate framework. For sample co-doped with V and Fe, three absorption bands centered at 216 nm, 264 nm, and 374 nm are visible, which resembles that of V-MCM-48. This indicates the introduction of Fe does not affect the coordination circumstance of V. Additionally, there may be an overlap in the 254 and 264 nm bands, implying that Fe may be also well incorporated into the silicate framework as the isolated tetrahedral coordination Fe^3+^ species.

#### 2.2.2. XPS

**Figure 5:**
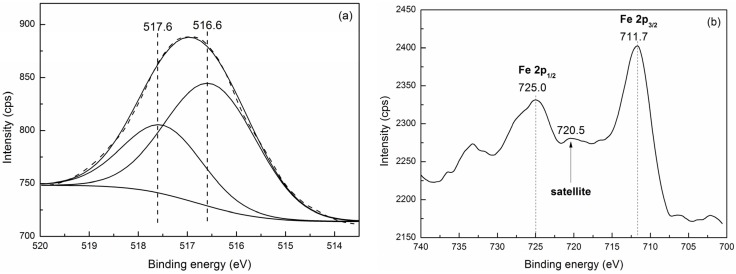
X-ray photoelectron spectra (XPS) of (**a**) V 2p_3/2_ and (**b**) Fe 2p from the calcined V-Fe-MCM-48 sample.

Because in the wide-angle XRD patterns peaks corresponding to the bulk V_2_O_5_ and Fe_2_O_3_ are absent, we further use XPS to evaluate the surface composition and the chemical states of vanadium and iron species. As shown in Figure 5a, the XPS spectrum of the V 2p region of the V-Fe-MCM-48 sample can be deconvoluted into two components centered at 517.6 eV (A) and 516.6 eV (B), respectively. The component A is in good agreement with the V 2p_3/2_ observed for V_2_O_5_ indicating that vanadium exists on the exposed surface as V^5+^. While the component B cannot be clearly assigned to V^4+^ or V^3+^ based solely on the absolute value of binding energy [50]. Alternatively, the component B is assigned based on the differences in the binding energies (ΔBE). It was reported that ΔBE between V^5+^ and V^4+^ is 0.7–1.0 eV, and 1.2–1.5 eV between V^5+^ and V^3+^. The ΔBE between the component A and B obtained from the present study is 1.0 eV suggesting that the component B corresponds to V^4+^, which is agree well with the UV-vis results. Furthermore, it is observed that the peak area of V^4+^ is approximately twice that of V^5+^, and as shown in Table 1, the content of bulk vanadium in V-Fe-MCM-48 sample determined by ICP is much higher than that of vanadium exposed on the surface obtained from XPS. These results indicate that most of vanadium atoms are doped into the silicate framework. Figure 5b is the XPS spectrum of the Fe 2p region of the V-Fe-MCM-48. The peaks at 711.7 eV and 725.0 eV correspond to Fe 2p_3/2_ and Fe 2p_1/2_, respectively. The existence of satellite peak centered at 720.5 eV suggests that iron exists as Fe^3+^ in the sample. The binding energy value of Fe 2p_3/2_ obtained here is much larger than that of pure Fe_2_O_3_, which has been reported between 710.6 and 711.2 eV [51,52]. This result indicates that the iron atoms are surrounded by silicon.

### 2.3. Hydrothermal Stability Test

**Figure 6:**
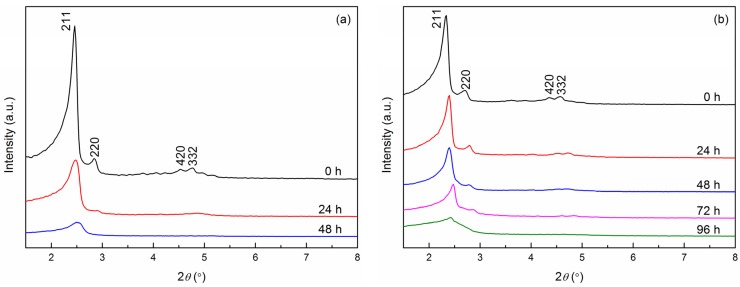
XRD patterns of the calcined (**a**) Si-MCM-48 and (**b**) V-Fe-MCM-48 samples after hydrothermal treatment at 110 °C for different time.

The hydrothermal stability of calcined Si- and V-Fe-MCM-48 samples treated at 110 °C for different time are evaluated by XRD. As shown in Figure 6a, the Si-MCM-48 still exhibits the (211) peak after treated for 24 h, whereas the (220) peak was not pronounced and the (420) and (332) peaks fully disappeared indicating that mesostructured turns to be less ordered. After 48 h treatment, the (220) peak fully disappears and the (211) peak is getting weak and broad implying that the 3D cubic mesostructure collapses to some extent. Therefore, the Si-MCM-48 exhibits a poor hydrothermal stability. This finding is probably due to the formation of hydrogen bonds between water molecules and the O atoms in Si-O-Si, which weakens the interaction between Si and O atoms and causes a breakage of Si-O-Si bonds. Consequently, some Si atoms may be detached from the framework, which brings some defect or collapse in the framework. For V-Fe-MCM-48 recorded in Figure 6b, the (220), (420) and (332) peaks are still visible after the treatment of 24 h indicating that the 3D cubic mesostructure of MCM-48 maintains highly ordered. The (420) and (332) peaks do not disappear until treated after 48 h. After further treatment of 72 h, the (220) peak is getting much more weak and almost invisible after 96 h treatment when the (211) peak is weak and broad. This implies that the 3D cubic mesostructure collapses.

**Figure 7:**
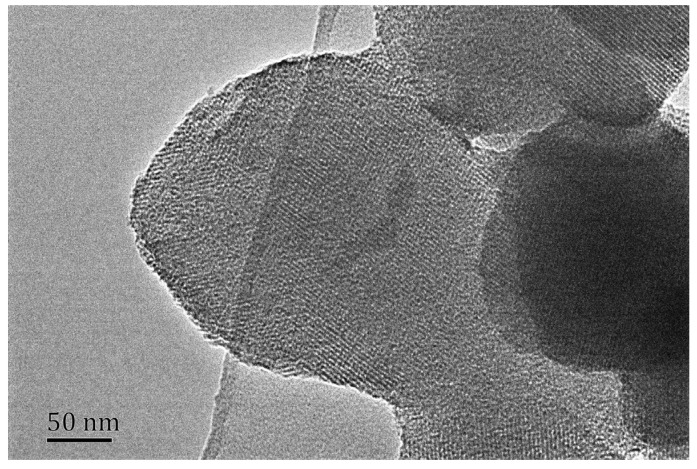
TEM image of the V-Fe-MCM-48 sample after 72 h hydrothermal treatment.

To investigate the extent of loss of the ordered mesoporous structure with hydrothermal treating time, TEM of V-Fe-MCM-48 treated for 72 h has been done. As displayed in Figure 7, the sample treated for 72 h shows much more disordered mesoporous structure when compared with that of the V-Fe-MCM-48 with no treatment exhibited in Figure 3b, which is consistent well with the small-angle XRD patterns. Furthermore, the particles severely aggregated which may be due to the polymerization of surface Si-OH groups.

**Figure 8:**
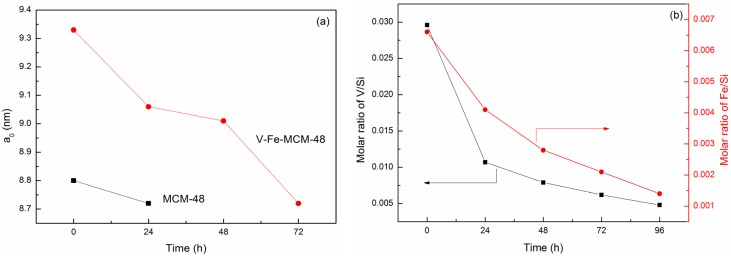
Development of (**a**) the unit cell parameter a_0_ of MCM-48 and V-Fe-MCM-48 and (**b**) content of V and Fe in V-Fe-MCM-48 with the time of hydrothermal treatment.

Also, in the hydrothermal stability test section the d-values are shifting with treatment time. For a deeper insight into the structural behavior, some structural parameters of the hydrothermally treated MCM-48 and V-Fe-MCM-48 samples at different time are given in Table 2, and a fitting of the data are shown in Figure 8. As displayed in Figure 8a, both the unit cell parameter a_0_ of MCM-48 and V-Fe-MCM-48 decrease with treatment time, suggesting a shrinkage of pore wall caused by the breakage of Si-O-Si bonds. Moreover, the a_0_ of V-Fe-MCM-48 decreases more significantly than that of MCM-48, together with a decreasing of metal content to some extent with the treating time found in Figure 8b, implying that most V and Fe species have been leached out from the silicate framework, which could be one of the reasons why the mesoporous structure becomes more disordered with time. Nevertheless, the V and Fe are still detected by ICP after 96 h treatment when the mesoporous structure has collapsed. Thus we proposed that doping heteroatoms played an important role in stabilizing the silicate framework at the prior hydrothermal treatment, and the mesoporous structure collapsed after 96 h may be due to the breakage of Si-O-Si bonds caused by the hydrogen bonds between water molecules and the O atoms in Si-O-Si bonds, which is the same as that of MCM-48.

**Table 2:** Structural parameters of the Si- and V-Fe-MCM-48 samples with different treatment time.

Time (h)	MCM-48		V-Fe-MCM-48			
	d_211_ (nm)	a_0_^a^ (nm)	V/Si ^a^ (Molar Ratio)	Fe/Si ^b^ (Molar Ratio)	d_211_ (nm)	a_0_^a^ (nm)
0	3.59	8.80	0.0296	0.0066	3.81	9.33
24	3.56	8.72	0.0107	0.0041	3.70	9.06
48	–	–	0.0079	0.0028	3.68	9.01
72	–	–	0.0062	0.0021	3.56	8.72
96	–	–	0.0048	0.0014	–	–

Notes: ^a^ Unit cell parameter: a_0_ = 6^1/2^d_211_; ^b^ ICP results.

## 3. Experimental Section

### 3.1. Materials

Tetraethylorthosilicate (TEOS) was purchased from Sinopharm Chemical Reagent Co., Ltd.; cetyltrimethylammonium bromide (CTAB), ammonia solution (25–28 wt %), ethanol, hydrogen peroxide (H_2_O_2_, 30 wt %), vanadium metavanadate (NH_4_VO_3_) and iron nitrate nonahydrate (Fe(NO_3_)_3_•9H_2_O) were purchased from Nanjing Chemical Reagent Co., Ltd. (Nanjing, China).

### 3.2. Synthesis of the Samples

The V-Fe-MCM-48 was synthesized via one-step hydrothermal method under precisely control, using CTAB as structure-directing agent, and EtOH as cosolvent. The reaction was carried out at room temperature. The molar ratio composition of the reaction mixture was 1.0TEOS:0.3CTAB:60EtOH:10NH_3_:0.04V:0.01Fe:410H_2_O, and the obtained sample was noted as V-Fe-MCM-48. A typical preparation of V-Fe-MCM-48 is as follow: 0.0432 g NH_4_VO_3_ and 0.0381 g Fe(NO_3_)_3_•9H_2_O were dissolved in 10 mL deionized water, respectively. Meanwhile, 1.0125 g CTAB was dissolved in 50 mL deionized water under mechanical stirring at 170 rpm, followed by adding 32.4 mL EtOH and 6.3 mL 25–28 wt % ammonium solution orderly. When complete dissolution, the NH_4_VO_3_ solution and Fe(NO_3_)_3_ solution were added dropwise successively to the template solution under mechanical stirring at 450 rpm. After 15 min stirring, 2.1 mL TEOS was added dropwise to the above mixture solution, and kept on being further stirred for 2 h. Then the synthesized gel was placed in the teflon-lined autoclave and aged at 100 °C for 24 h. The sample was washed with deionized water and EtOH till neutral, and dried at 70 °C in air for 10 h. The as-synthesized product was calcined at 550 °C in a flow of air for 6 h at a heating rate of 1 °C/min to remove the template. For comparison, Si-MCM-48 samples were also synthesized using the same method.

### 3.3. Characterization

The XRD patterns of the samples were collected with Smartlab TM 9 KW (Rigaku Corporation, Tokyo, Japan) equipped with a rotating anode and Cu Kα radiation (λ = 0.154178 nm).

Nitrogen adsorption-desorption isotherms were measured on a BELSORP-MINI analyzer (BEL Japan, Osaka, Janpan) at 77 K. Before the measurements, calcined samples were degassed in vacuum at 200 °C for 2 h. Surface areas were calculated using the BET equation and pore size distributions were obtained by the Barrett-Joyner-Halenda (BJH) method using desorption branch data.

The vanadium content and iron content in the samples were determined using a PE Optima 2000DV (PerkinElmer, Waltham, MA, USA) Inductively Coupling Plasma emission spectrometer (ICP). The samples were completely dissolved in hydrofluoric acid before analysis.

Field emission scanning electron microscopy (FE-SEM) was performed on a Hitachi S4800 Field Emission Scanning Electron Microscopy (Hitachi, Tokyo, Janpan).

Transmission electron microscopy (TEM) images were recorded on a JEM-2010 EX microscope (JEOL, Tokyo, Japan) operated at an accelerating voltage of 200 kV. The samples were crushed in A.R. grade ethanol, and the resulting suspension was allowed to dry on carbon film supported on copper grids.

Diffuse reflectance UV-vis spectra was recorded by a Lambda 950 spectrophotometer (PerkinElmer, Waltham, MA, USA) in the range of 200–800 nm with Ba_2_SO_4_ as reference.

The X-ray photoelectron spectra (XPS) were conducted on a PHI 5000 Versa Probe X-ray photoelectron spectrometer (ULVAC-PHI, Kanagawa, Japan) equipped with Al Karadiation (1486.6 eV). The C1s peak at 284.6 eV was used as the reference for binding energies.

### 3.4. Hydrothermal Stability Test

The hydrothermal stability test was carried out as followed. Two grams of calcined sample was added to a teflon-lined autoclave with 100 mL distilled water. Then the autoclave was placed to a drying oven and treated at 110 °C for different time (24, 48, 72, and 96 h). The sample was recovered by filtration and dried at 70 °C in air for 10 h. To evaluate the hydrothermal stability, XRD was used to characterize the structure.

## 4. Conclusions

In this paper, we have successfully synthesized vanadium and iron co-doped MCM-48 with spherical morphology, narrow pore size distribution and high specific surface area by one-step hydrothermal method. Most of the vanadium and iron species were incorporated into the silicate framework as tetrahedral V^4+^ and Fe^3+^ species, respectively. The introduction of iron species reduced the particle sizes and increased the specific surface area significantly of V-MCM-48 with almost the same amount of vanadium species. Moreover, the hydrothermal stability of Si-MCM-48 improved by co-doping the silicate framework with V and Fe. We offer a direct and simple method to produce MCM-48 with remarkable hydrothermal stability, which promotes its usage in hot water and aqueous solution.
